# The Impact of COVID-19 Epidemic on the Development of the Digital Economy of China—Based on the Data of 31 Provinces in China

**DOI:** 10.3389/fpubh.2021.778671

**Published:** 2022-01-27

**Authors:** Aidi Xu, Fangbin Qian, Chih-Hung Pai, Na Yu, Pan Zhou

**Affiliations:** ^1^School of Hospitality Management, Zhejiang Yuexiu University, Shaoxing, China; ^2^Center for International Education, Philippine Christian University, Manila, Philippines; ^3^College of Business and Economics, Sejong University, Seoul, South Korea

**Keywords:** COVID-19, digital pneumonia, China economy, sustainability, uncertain public risks

## Abstract

This study investigates the impact of coronavirus disease 2019 (COVID-19) on economic development of China by measuring the HP financial index as an alternative variable of the digital economy. This study shows that economy of China developed further with the dissemination of COVID-19. Furthermore, the digital economy increased the level of economic development more prominently at the onset of COVID-19 pandemic. Moreover, an analysis of regional heterogeneity reveals that the eastern region maintained economic stability through its digital economy during COVID-19, while the central region improved its digital economy during COVID-19 pandemic. Although the economically underdeveloped western region has not suffered too seriously from COVID-19 pandemic, considering the sustained impact of disease and the uncertainty of its transmission speed, the region should vigorously develop its digital economy to manage public risk.

## Research Background and Literature Review

The news about coronavirus disease 2019 (COVID-19) broke out in Wuhan, China in December 2019 and spread to the whole country at an extremely rapid rate. As of 29 August, 2021, the number of confirmed cases in China has reached 151,122,891 million. The emergence of COVID-19 outbreak has had a serious negative impact on economy of China. The growth rate of gross domestic product (GDP) of China decreased by 5.25% in 2019 compared to that in 2020 and the impact on the secondary and tertiary industries is the greatest. According to the data of the National Bureau of Statistics, the GDP of secondary industries shows negative growth from 2019 to 2020. Despite the news of the outbreak of COVID-19 rapidly spreading in China, the impact on its economy has not been so severe owing to the vigorous development of the digital economy.

### Coronavirus Disease 2019 Pandemic and the Digital Economy

Coronavirus disease 2019 pandemic has seriously affected secondary and tertiary industries in China. China has essentially overcome the short-term impact of COVID-19. The negative impact on the public will endure; however, it will simultaneously increase development opportunities of enterprises. Zhou et al. (1) and others pointed out that the digital economy can help in economic recovery and development of China through the dual path of efficiency improvement and innovation and development, even though COVID-19 pandemic persists. For example, fresh food e-commerce website, Hema Xiansheng, illustrates the premise that the service industry can be supported by digital technology, save production costs and operating costs, and improves resource allocation efficiency. Its continuous innovation trend conforms to the opportunity derived from COVID-19 pandemic. Ma (2) indicated that the emergence of COVID-19-related information has advanced the development of e-commerce to a certain extent and the subsequent development of e-commerce has played a huge role in driving economic growth. According to statistics on e-commerce, the proportion of GDP increased from 20.8 to 34.8%. The development of e-commerce helps to eliminate the negative impact of COVID-19 pandemic on economic development and plays a “complementary” role in the real economy. Lou (3) posits that the emergence of the digital economy has strengthened cooperation between China and Latin American countries in the context of the information shared about COVID-19. It has played a vital role in resolving the downward pressure on the bilateral economic and trade cooperation affected by COVID-19 pandemic and in deepening the cooperation between China and Latin America, thus strengthening the “one belt and one way” cooperation and acquiring a higher level and wider area after the outbreak of COVID-19. Cheng (4) revealed that COVID-19 pandemic has severely impacted traditional industries such as manufacturing and tourism in China, but the digital economy has strongly developed. Several traditional economic operations, such as offices and educational institutions, have transformed into the digital economy enterprises, using the cloud and online activities, which have stimulated the development of digital technologies and infrastructure such as artificial intelligence and 5G in China.

Lu (5) presented the opportunities and challenges of the digital economy during COVID-19 pandemic. He believes that during COVID-19 pandemic, the demand for online services has increased and, therefore, traditional enterprises need to achieve digital transformation, state governance needs to be smart, and the public health sector needs to be transformed using smart technology, promoting the development of the digital economy. However, simultaneously, there are great obstacles to the development of the digital economy, including, slow intelligent transformation in the manufacturing sector, insufficient core technology innovation, and insufficient construction of new infrastructure. To make the digital economy better serve economy of China, we should concentrate efforts on solving the current problem. Economic cooperation of China has been restored through the digital platform. The “China-ASEAN Digital Economic Cooperation Year” has boosted the economic and trade cooperation between the two sides, providing a very convenient platform to develop digital economic cooperation and the digital economy along the “one belt and one road” area (6).

### Digital Economy and Level of Economic Development

Wei (7) pointed out that the development of the digital economy has penetrated daily travel patterns of residents and the development of the digital economy of China has maintained a growing trend with rapid development and great potential. With the development of the internet in the 21st century, the world entered the information age and many industries supervise and manage their business links based on the digital economy. Its diversified-related businesses are also expanding rapidly and its business functions are becoming increasingly powerful. In addition, he indicated that the development of the digital economy must improve laws and policies, support its upstream and downstream industries from many aspects, and promote the digital economy to increase economic construction. Wu (8) concurred through empirical analysis that the digital economy is the general trend of future development and an effective strategy to promote economic growth. By changing the information flow, the transaction costs of buyers and sellers are reduced, which change consumption patterns and promote economic growth in China. Furthermore, Li et al. (9) analyzed the coupling relationship between the two groups of data of “e-commerce development level—high-quality economic development” in 31 provinces and cities and concluded that there is a complex correlation between them. This study shows that the degree of coupling between the level e-commerce development and high-quality economy in the eastern region is better. The central and western regions can refer to the development of e-commerce in the eastern region and emulate it. Therefore, it is necessary to develop the digital economy and lay foundations for e-commerce growth.

In addition, Mclean (10) studied the encouragement of the digital economy for economic growth by applying the successful DeLone and McLean model to digital economic measurement. They indicated that the dimensions of the digital economy success and the choice of measures should depend on the objective and background of empirical investigation, but tested and proven measures should be used as far as possible. It is important to measure the possible interactions among the dimensions of success to isolate the influence of independent variables on one or more of them such as individuals, groups, organizations, and industries. Walden (11) pointed out in an article on the research direction of economics and the digital economy that the digital economy will eventually change business behavior and structure as we know it. They described a series of interesting developments and innovations in emerging industries. Furthermore, they expressed that with the development of the digital economy, the gradual improvement of information has made consumers more actively inclined toward lower-priced products. This user tendency increases during product promotion activities because users have established lower price expectations.

Thus, this study proposes hypothesis 1.

H1: The digital economy has a positive impact on economic development level of China.

### Coronavirus Disease 2019, the Digital Economy, and Levels of Economic Development

The development of the digital economy has offset the negative impact of COVID-19 pandemic on economy of China. Wang (12) and others systematically expounded the opportunities for the digital economy industry development of China during COVID-19 pandemic from the perspective of industrial transformation and upgrading. They achieved this by scrutinizing the relationship between COVID-19 pandemic situation and the economy and analyzed COVID-19 pandemic as a catalyst of the development of the digital economy industry from dual perspectives of market and government. It is stressed that the digital economy has played an active role under the outbreak of COVID-19 in four aspects: expanding social distance, monitoring population mobility, enhancing information transmission and public policy efficiency, and enriching the form of stimulating the economy.

Researchers (1, 2, 4, 13) discussed the mechanism on how COVID-19 affected the development of the digital economy in China. First, epidemic prevention provides new space for the rapid development of the digital economy. 5G communication, block chain, artificial intelligence, and other new generation information technologies play an important role in the safe and orderly resumption of work and production. 5G provides effective network guarantee for the smooth operation of remote services such as online medical treatment, video conference, and online office. Second, manufacturing enterprises actively rely on the digital platform to gather resources and accelerate the overall resumption of work and production of the manufacturing industry. For example, Haier Group has established a medical material information sharing resource aggregation platform to provide epidemic information management and analysis services. Some companies provide free remote video communication, remote collaborative office, high-definition (HD) video conference system, and other services. Face recognition, intelligent infrared thermometry, big data analysis, and comparison technology are also important means to ensure effective personnel control after returning to work. Third, it has stimulated healthy and green consumption, further expanded online consumption content, consumption forms, and new market space in terms of online education, online office, online medical treatment, and digital marketing. With the iteration of consumption habits of residents, the traditional industry development model and interest chain may be changed, which will promote the continuous enrichment of online consumption content and scenes, the stickiness to online channels, and the continuous growth of the digital economy.

Studies reveal that COVID-19 pandemic provides many opportunities for the rapid development of the digital economy of China. The driving force is mainly the changes in market demand and the improvement of the governance capabilities of the government during COVID-19 pandemic. Implementing structural reforms to “enhance motivation” is an important response strategy for the government to transform COVID-19 pandemic crisis into a new driving force for industrial development and to cultivate long-term economic development potential.

Furthermore, Li et al. (9) and others hold that the platform economy is the most important business mode and economic form in the digital economy era. The influence of the development of the digital economy during COVID-19 on economy of China is analyzed from demand and supply perspectives. The study shows that in the short term, platform enterprises have performed well during COVID-19 pandemic, but the overall platform economy has been negatively impacted due to some factors such as macroenvironment, industrial chain, and supply chain; however, overall, COVID-19 pandemic has accelerated the development processes of platform economies. Therefore, to promote its healthy development after COVID-19 pandemic, we should strengthen the support for platform enterprises in science, technology, and finance and pay attention to information security.

Luo and Lu (14) and others posited the use of the digital technology to accelerate the group transformation and upgrading of small and medium-sized enterprises (SMEs) from a microperspective to enhance the level of economic development of China. This study shows that the digital technology provides the direction and path for SMEs to accelerate their transformation and upgrade in the fight against COVID-19 pandemic, which includes five aspects: technical sources, financial support, industrial collaboration, market application, and talent team building.

Moreover, Qian et al. (15) and others have summarized the economic stimulus plans of countries around the world in the context of COVID-19 pandemic and found that the development direction always revolves around two keywords, “digitalization” and “greening” and proposed that the digital economy can be combined with the green economy. With respect to coordinated development, on one hand, the digital economy can effectively promote the green transformation of the global economy. On the other hand, the green economy can help the digital economy to achieve green, low-carbon, and sustainable development. Therefore, best strategy of China for economic development in the postpandemic period is to include both the economy and digital economy in the policy, which will be able to furthering maximize its role in economic recovery of China after COVID-19 pandemic.

Analysis by Joseph et al. (16) shows that COVID-19 is a great accelerator to speedily track existing global trends, namely, embracing modern emerging technologies, leading to changes in lifestyle, working patterns, and business strategies. Therefore, COVID-19 has evolved into a catalyst, which has led to the increased application of the digital economy in organizations and offices and has brought foreseeable and unforeseen opportunities, challenges, and costs. Yushan et al. (17) and others believe that the global spread of COVID-19 has significantly impacted healthcare, social life, and the economy. However, the digital economy plays a vital role in realizing ubiquitous and accessible digital health services during COVID-19 pandemic and in preventing the recurrence of COVID-19 in the postpandemic era. Moreover, Mustafa and Mohamed (18) explores modern medical technology and artificial intelligence development trends during COVID-19 pandemic. Study indicates that interventions in the digital economy have strengthened the response to COVID-19 and magnified the role of medical imaging in the information about the related crisis and gives medical professionals access to noncontact care opportunities. Study shows that COVID-19 information platform must be adjusted rapidly with the support of digital technology. With the new “normal” situation consumers and retailers are finding themselves in, the development of the digital economy is crucial to recovery of China.

Thus, this study proposes hypothesis 2.

H2: During COVID-19 outbreak, the development of the digital economy increased level of economic development of China.

Previous study on COVID-19, the digital economy, and levels of economic development generally focus on theoretical study and policy suggestions and seldom discuss the relationship among them through empirical study. Therefore, this study examines 31 provinces in China as study objects to study the impact of the digital economy on the level of economic development of China during COVID-19 pandemic.

### Digital Economy Index System and Measurement

The definition of the digital economy by scholars mainly includes two aspects: (1) with respect to form, the digital economy is an economic form that guides and realizes the rapid optimization and regeneration of resources and achieves high-quality economic development; (2) at the technical level, the digital economy includes big data. A collection of emerging technologies includes cloud computing, the Internet of Things, block chain, artificial intelligence, and 5G communication. At the application level, “new retail” and “new manufacturing” are both typical representatives. According to the definition of the digital economy, it is difficult to quantify it. Therefore, this study uses the digital inclusive financial index used by Guo et al. (19) to represent the digital economy. The digital inclusive financial index is a comprehensive definition based on the connotation and characteristics of the digital economy. Each index and dimension reflects a certain perspective of the digital economy. Table 1 shows specific index system construction.

**Table 1:** Digital economy index system.

**First-level dimension**	**Secondary** **dimension**	**Specific indicators**
		Number of Alipay accounts per 10,000 people
Coverage	Account coverage	Percentage of Alipay users according to their cards
		Average number of bank cards bound to each Alipay account
		Per capita payment
	Payment business	Amount paid per capita
		High frequency (50 times or more per year) active users accounted for one time or more uses per year
		Per capita purchases of Yu'e Bao
	Money fund business	Per capita purchase amount of Yu'e Bao
		Number of people who purchased Yu'e Bao, per 10,000 Alipay users
		The number of users with internet consumer loans per million Alipay adult users
	Personal consumption loans	Per capita loans
		Per capita loan amount
		The number of small and micro internet business loan users per million Alipay adult users
Investment business	Small and micro business operators	Average number of loans per household for small and micro-operators
		Average loan amount of small and micro-operators
		Number of insured users per million Alipay users
	Insurance business	Insurance per capita
		Insurance amount per capita
		Number of people participating in internet investment and wealth management per 10,000 Alipay users
	Investment business	Investment per capita
		Investment amount per capita
		Per capita credit calls for natural persons
	Credit business	Number of users using credit-based services per Alipay user (including financial, accommodation, travel, and social)
	Mobility	Proportion of mobile payments
		Proportion of mobile payment amounts
	Affordability	Average loan interest rate for small and micro-operators
		Average personal loan interest rate
		Credit
		Proportion of Huabei's payment amount
Degree of digitization	Creditization	Proportion of the number of Credit Sesame free deposits (compared to all cases where a deposit is required)
		Percentage of Credit Sesame free deposits (compared to all cases where deposits are required)
		Percentage of user QR code payment
	Facilitation	The proportion of the amount paid by the user's QR code

*QR, quick response*.

It is difficult to measure the digital economy using a single index, so we select indicators from different dimensions and use the entropy method to calculate the comprehensive score of the digital economy. The steps of the entropy method are as follows:

(1) Construct the original evaluation matrix: assuming there are *t* years, *a* province, and *b* evaluation indicators, the original evaluation matrix is *X* = (*x*_θ*ij*_). j = 1,..., b. For example, *X* = (*x*_θ*ij*_) in the matrix represents the *jth* index value of province *i* in the θ*th* year of the system.
(2) Dimensionless processing of indicators, for positive indicators, using formula 1, for negative indicators, using formula 2, to avoid the occurrence of zero values, this study standardizes each indicator and shifts 0.1 units.

(1) $$\begin{array}{l} M_{\theta ij}=\frac{x_{\theta ij}-\min_{j}\left(x_{\theta ij}\right)}{\max_{j}\left(x_{\theta ij}\right)-\min_{j}\left(x_{\theta ij}\right)}+0.1 \end{array}$$

(2) $$\begin{array}{l} M_{\theta ij}=\frac{\max_{j}\left(x_{\theta ij}\right)-x_{\theta ij}}{\max_{j}\left(x_{\theta ij}\right)-\min_{j}\left(x_{\theta ij}\right)}+0.1 \end{array}$$

(3) Calculate the proportion of each index in the corresponding sample. See (3) for the formula:

(3) $$\begin{array}{l} p_{\theta ij}=\frac{M_{\theta ij}}{\underset{j}{\sum }M_{\theta ij}} \end{array}$$

(4) Calculate the entropy value and difference index of the index. See (4) for the formula:

(4) $$\begin{array}{l} e_{j}=k\underset{j}{\sum }p_{\theta ij}\ln\;\left(p_{\theta ij}\right),\;g_{i}=1-e_{i} \end{array}$$

Where $k=\frac{-1}{\ln\;\left(nt\right)}$, where *n* is the number of research samples and *t* is the year interval.

(5) Calculate the weight of each indicator and calculate the comprehensive score, see formula (5), where *w*_*j*_ is the weight of indicator *j* and *DE*_θ*i*_ is the comprehensive score of the *ith* province in the digital economy of the θ year. The specific scores are shown in Table 2.

(5) $$\begin{array}{l} w_{j}=\frac{g_{j}}{\overset{b}{\underset{j=1}{\sum }}g_{j}}\;DE_{\theta i}=\overset{b}{\underset{j=1}{\sum }}w_{j}M_{\theta ij} \end{array}$$

The results in Table 2 reveal that the level of digital economic development in 16 provinces, municipalities, and autonomous regions in China are above the average, indicating that during the outbreak of COVID-19, level of the digital economic development of China is relatively balanced and relative to 2019, there are varying degrees of growth, indicating that the digital economic industry is indeed survivable during the outbreak of COVID-19.

**Table 2:** Results of the entropy method.

**Province**	**2019 digital economy total score**	**2020 digital economy total score**	**Growth rate (%)**
Beijing	399.00	417.88	4.73
Tianjin City	344.11	361.46	5.04
Hebei province	305.06	322.70	5.78
Shanxi province	308.73	325.73	5.51
Inner Mongolia autonomous region	293.89	309.39	5.27
Liaoning province	311.01	326.29	4.91
Jilin province	292.77	308.26	5.29
Heilongjiang province	292.87	306.08	4.51
Shanghai	410.28	431.93	5.28
Jiangsu province	361.93	381.61	5.44
Zhejiang province	387.49	406.88	5.00
Anhui province	330.29	350.16	6.02
Fujian province	360.51	380.13	5.44
Jiangxi province	319.13	340.61	6.73
Shandong province	327.36	347.81	6.25
Henan province	322.12	340.81	5.80
Hubei province	344.40	358.64	4.13
Hunan province	310.85	332.03	6.81
Guangdong province	360.61	379.53	5.25
Guangxi Zhuang autonomous region	309.91	325.17	4.92
Hainan	328.75	344.05	4.65
Chongqing	325.47	344.76	5.93
Sichuan province	317.11	334.82	5.58
Guizhou province	293.51	307.94	4.92
Yunnan province	303.46	318.48	4.95
Tibet	293.79	310.53	5.70
Shaanxi province	322.89	342.04	5.93
Gansu province	289.14	305.50	5.66
Qinghai province	282.65	298.23	5.51
Ningxia Hui autonomous region	292.31	310.02	6.06
Xinjiang Uygur autonomous region	294.34	308.35	4.76

## Empirical Research

### Variable Selection

#### Interpreted Variable

The variable explained in this study is the level of economic development, which is measured by per capita GDP. In the robustness test, the number of night lights is used to measure the level of economic development.

#### Explanatory Variables

The main explanatory variables in this study are the digital economy and COVID-19 pandemic. The data selection for the digital economy refers to the results of the entropy method explained above. For COVID-19 pandemic impact analysis literature, the explanatory variable usually uses the number of confirmed patients or the number of deaths as a measure of the severity of COVID-19 pandemic. This study selects the number of confirmed cases in 31 provinces in China as the explanatory variable.

#### Control Variable

In this study, the levels of urbanization, industrialization, foreign direct investment, and industrial structure upgradation are selected as control variables. The ratio of the resident population to the total area of each region in the current year is used to indicate the level of urbanization. The number of industrial enterprises above the scale in each region in the current year is used to measure the level of local industrialization. The amount of foreign direct investment is a measure of it. Moreover, the ratio of the tertiary industry total output value to the second industry total output value measures the level of industrial structure upgrading. Relevant data come from the provincial and municipal statistical yearbooks.

### Model Building

Based on the above choices of explanatory and control variables, this study establishes a multiple linear regression model to explore the direct impact of COVID-19 pandemic and the digital economy on level of economic development of China and the indirect impact of the digital economy on economic development level of China during COVID-19 pandemic. Logarithmically, the model is processed as follows:


(1) $$\begin{array}{l} \ln\;GDP_{it}=\alpha_{0}+\beta_{0}\ln\;DE_{it}+\beta_{1}\ln\;COV_{it}+\beta_{2}\ln\;SIZE_{it}\\ \;+\beta_{3}\ln\;CITY_{it}+\beta_{4}\ln\;FDI_{it}+\beta_{5}\ln\;ADV_{it}\\ \;+u_{i}+\zeta_{it} \end{array}$$

(2) $$\begin{array}{l} \ln\;GDP_{it}=\alpha_{0}+\beta_{0}\ln\;DE_{it}+\beta_{1}\ln\;COV_{it}+\beta_{2}\ln\;DE_{it}\\ \;*\ln\;COV_{it}+\beta_{3}\ln\;SIZE_{it}+\beta_{4}\ln\;CITY_{it}+\beta_{5}\ln\;FDI_{it}\\ \;+\beta_{6}\ln\;ADV_{it}+u_{i}+\zeta_{it} \end{array}$$

Gross domestic product represents the level of economic development, DE represents the digital economy, COV represents COVID-19 outbreak, FDI represents foreign direct investment, SIZE represents the level of industrialization, CITY represents the scale of the city, and ADV represents the advanced industrial structure. α_0_ is a constant and ζ_*it*_ is a random error term.

### Data Sources and Descriptive Statistics

Since this study uses the per capita GDP of each province, municipality, and autonomous region to measure the level of economic development, economic units have been treated as price reductions. Descriptive statistics for each variable are shown in Table 3.

**Table 3:** Descriptive statistics of variables.

**Variable**	**Mean value**	**Standard deviation**	**Minimum value**	**Maximum**
Gross domestic product (GDP)	11.08	0.39	10.40	12.01
Digital economy (DE)	5.80	0.10	5.64	6.07
COVID-19 outbreak (COV)	4.26	3.34	0	11.13
Industrialization level (SIZE)	1.834	1.197	−1.064	4.182
Urbanization level (CITY)	7.664	0.645	5.225	8.749
Foreign direct investment (FDI)	3.258	0.847	1.450	6.194
Advanced industrial structure (ADV)	4.624	0.580	3.230	6.193

According to the descriptive statistical results in Table 3, the average value of the level of economic development is 11.08 and the maximum and minimum values are 10.40 and 12.01, respectively. It is found that current economic development level of China is relatively overall balanced. The average value of the digital economy is 5.8 and the maximum and minimum values are 6.07 and 5.64, respectively, indicating that the overall level of the digital economy of China is relatively balanced and there is little difference in the digital economy investments among different regions. The average value of COVID-19 pandemic situation was 4.26 and the maximum and minimum values were 11.13 and 0, respectively. Except for Hubei province, the confirmed cases of COVID-19 are similar.

Observation of the descriptive statistical results of the control variables reveals that the average value of the industrialization level is 1.834 and the maximum and minimum values are 4.182 and −1.064, respectively, indicating that during COVID-19 pandemic, industries in some regions of China were severely affected. The average urbanization level is 7.664 and the maximum and minimum values are 8.749 and 5.225, respectively, indicating that level of urbanization of China is relatively balanced. The average value of FDI is 3.258 and the maximum and minimum values are 8.749 and 5.225, respectively, indicating that overall level of foreign investment attraction of China is high. The average value of industrial structure upgrading is 4.624 and the maximum and minimum values are 6.193 and 3.230, respectively, indicating that the production scale and technology level of some industries are still at a low level.

## Empirical Analysis

### Direct Impact of COVID-19 Pandemic and the Digital Economy on Level of Economic Development of China

Table 4 shows the impact of COVID-19 pandemic and the digital economy on level of economic development of China. The coefficient of the digital economy is significantly positive at the 1% level and shows an increasing trend, which indicates that the development of the digital economy significantly stimulated economy of China during COVID-19 pandemic and the coefficient of the digital economy is larger than at the beginning of COVID-19 pandemic in 2019, which verifies hypothesis 1. Moreover, the coefficient for COVID-19 information is significantly negative at the 1% level and the coefficient has always shown an increasing trend. This shows that after the outbreak of the news about COVID-19 in Wuhan, it spreads swiftly to the whole country and the negative impact on level of economic development of China started intensifying.

**Table 4:** The direct impact of coronavirus disease 2019 (COVID-19) pandemic and the digital economy on the level of economic development of China.

	**2019**	**2020**
Digital economy (DE)	0.2156*** (2.94)	0.6757*** (3.49)
COVID-19 outbreak (COV)	−0.0124*** (−0.31)	−0.1749*** (−0.85)
Industrialization level (SIZE)	1.4152*** (5.23)	1.5124*** (5.67)
Urbanization level (CITY)	0.2524*** (2.34)	0.1641*** (2.17)
Foreign direct investment (FDI)	−0.153* (−1.21)	−0.2231* (−1.64)
Advanced industrial structure (ADV)	1.5124* (6.21)	2.2241* (6.45)
Constant	−3.5355*** (−8.55)	−4.5524*** (−8.96)

*** and * represent significant level at 1%, 5% and 10% respectively.

The results of the control variables indicate that the coefficient of the level of industrialization is significantly positive at the 1% level and the promotion effect is significant. Moreover, the coefficient for the level of urbanization is significantly positive at 1% probably because it is a solid foundation for economic development with an improvement of infrastructure. FDI is significantly negative at the level of 10%, which may be due to crowding out the domestic investment level, the decline of the overall domestic investment level, and insufficient innovation ability, which hinders level of economic development of China. The level of industrialization is significantly positive at 1%, which means that economic development of China cannot be separated from its development of industrialization. With COVID-19 pandemic, industry has been hit hard and the digital economy should be urgently developed to transform industry intelligence and stimulate economic development of China.

### Indirect Impact of COVID-19 Outbreak and the Digital Economy on Level of Economic Development of China

Table 5 presents the indirect impact of the information spread about COVID-19 and the digital economy on the level of economic development in China. This result reveals that the crossover between COVID-19 outbreak and the digital economy at the 1% level is significantly positive and shows an increasing trend. On one hand, it proves that the development of the digital economy has increased level of economic development of China. On the other hand, it proves that the positive effect of the digital economy on level of economic development of China gradually strengthened while COVID-19 was circulated. Observing the coefficients of the novel coronary pneumonia pandemic and the digital economy, we can see that compared with the coefficients of COVID-19 pandemic and the digital economy in Table 4, the coefficients of explanatory variables in Table 5 are significantly unchanged, but are only slightly reduced. This proves that the digital economy plays an intermediary role during COVID-19 pandemic, affecting the level of economic development.

**Table 5:** Indirect impact of COVID-19 pandemic and the digital economy on level of economic development of China.

	**2019**	**2020**
Digital economy (DE)	0.1864*** (1.87)	0.4987*** (2.95)
COVID-19 outbreak (COV)	−0.0116*** (−0.12)	−0.1975*** (−0.45)
The intersection of the digital economy and	0.3646*** (3.53)	1.5393*** (6.238)
the COVID-19 pandemic (DE*COV)		
Industrialization level (SIZE)	1.4241*** (5.21)	1.5244*** (5.52)
Urbanization level (CITY)	0.2212*** (2.22)	0.1241*** (2.08)
Foreign direct investment (FDI)	−0.1241* (−1.09)	−0.2241* (−1.32)
Advanced industrial structure (ADV)	1.5553* (6.11)	2.2767* (6.34)
Constant	−3.5685*** (−8.12)	−4.5457*** (−8.24)

*** and * represent significant level at 1%, 5% and 10% respectively.

By observing the coefficients of the control variables, we can see that they are reduced to varying degrees on the premise of unchanged significance, which indicates that the effect of COVID-19 on the digital economy has reduced the influence of other variables on the level of economic development, which further illustrates the important role of the digital economy in restoring level of economic development of China during COVID-19 pandemic.

### Regional Analysis

An analysis of regional heterogeneity is given in Table 6. From it, we can see that the eastern region has the highest level of digital economic development, the central region is second, and the western region has the lowest level, though it is above the 1% significance level. The results show that the eastern region with its higher level of economic development is more suitable to bring the effect of the digital economy into play and it maintains its own economic stability through the digital economy during COVID-19 outbreak. COVID-19 pandemic in the central region is too serious because it is the origin of COVID-19 pandemic. However, compared with the eastern region, the central region has better developed the role of the digital economy during COVID-19 pandemic such as through online office working and online education. The government judged the situation in the central region the first time and chose the most suitable strategy for its development. Therefore, COVID-19 pandemic did not cause drastic economic losses to China and the growth of its digital economy revived economic development of China. The western region is economically underdeveloped, but the unique geographical location of its provinces, municipalities, and autonomous regions protects it from extensive damage by COVID-19 pandemic. However, considering the sustained impact of COVID-19 and the uncertainty of its spread, the western region should vigorously develop its digital economy to face uncertain public hazards.

**Table 6:** Regional heterogeneity of the impact of COVID-19 and the digital economy on level of economic development of China.

	**East**	**Middle**	**West**
Digital economy (DE)	0.7612*** (2.87)	0.5212*** (1.95)	0.2153*** (1.24)
COVID-19 outbreak (COV)	−0.165*** (−0.12)	−0.345*** (−0.45)	−0.021*** (−0.08)
The intersection of digital economy and	0.7831** (3.53)	0.7952*** (6.238)	0.2415* (2.425)
COVID-19 pandemic (DE*COV)			
Industrialization level (SIZE)	1.5055*** (5.45)	1.4718*** (5.21)	1.2405*** (4.92)
Urbanization level (CITY)	0.3058*** (3.53)	0.3019*** (3.21)	0.3176*** (3.16)
Foreign direct investment (FDI)	−0.3591*** (−2.52)	−0.4061*** (−2.41)	−0.2860*** (−2.15)
Advanced industrial structure (ADV)	2.341*** (3.24)	2.4129*** (3.41)	1.7010*** (2.94)
Constant	−3.5685*** (−8.12)	−4.5457*** (−8.24)	−2.4214*** (−6.21)

***, **, and * represent significant level at 1%, 5% and 10% respectively.

## Conclusion and Policy Recommendations

Through the empirical study of the direct impact of COVID-19 pandemic and the digital economy on level of economic development of China, the findings revealed that the development of the digital economy considerably increased economic development of China and compared with the early pandemic of 2009, the digital economy enhanced economic development during current COVID-19 pandemic. Furthermore, with the outbreak of the novel coronary pneumonia virus in Wuhan, the speed of transmission to the whole country was accelerated and severely impacting economic development of China.

Through an empirical analysis of the indirect impact of COVID-19 and the digital economy on level of economic development of China, this study found that progress of the digital economy during COVID-19 pandemic enhanced level of economic development of China and compared to previous pandemics, the positive effect of the digital economy gradually strengthened. The digital economy played an intermediary role during COVID-19, affecting level of economic development of China.

An analysis of regional heterogeneity indicates that the eastern region with a higher level of economic development is more suitable for the digital economy in terms of capital and technology and has maintained its economic stability through the digital economy during COVID-19 outbreak. The central region is excessively affected because it is where COVID-19 originated. However, compared with the eastern region, the central region has greater success with the digital economy such as through online office work and online education. The government judged the situation of the central region at the beginning of COVID-19 pandemic and chose the most suitable strategy for it. Therefore, the novel coronary pneumonia pandemic has not caused serious economic losses to China. The rise of the digital economy has fortified the restoration of economic development of China. Though economically underdeveloped, the unique geographical location of the western region alleviated the provinces, municipalities, and difficulties of autonomous regions during COVID-19 pandemic. However, the lasting effects and the uncertainty of COVID-19 pandemic with respect to its swift spread, it requires the western region to develop the digital economy to face possible public endangerment. In view of the conclusions drawn in this study, the following policy suggestions are proposed:

(1) Formulate macropolicies: we must discern and influence the domestic and international trends of COVID-19 pandemic, steadily coordinate and promote various domestic economic and social development postpandemic initiatives, pay close attention to the development of COVID-19 pandemic, and actively plan response measures. The central level must formulate economic development policies such as fiscal policies, monetary policies, and industrial policies after COVID-19 pandemic and fully consider the long- and short-term impact of COVID-19 outbreak on economic development of China. To give full play to the leading role of fiscal policy, the Ministry of Finance, together with other departments, shall propose supporting policies to stabilize economic and social development and the relevant departments shall cooperate in their implementation. All the levels of financial departments should prioritize the financial advantage of all the levels and localities and provide discounts on special loans to key enterprises for COVID-19 pandemic prevention and control from the People's Bank of China.
(2) Policy formulation for the digital economy: there is need to transform effective short-term pilot policies of the digital economy into long-term policies to consider “digital industrialization, industrial digitalization, and digital governance” as the main line of development, to speed up the upgrade of the “Guiding Opinions on Accelerating the Development of Digital Economy” in various provinces and to promote pilot policies conducive to the development of the digital economy. COVID-19 pandemic has further highlighted the importance and vulnerability of SMEs in employment and in economic development of China. Some short-term government policies are required, at all the levels, to support the development of SMEs and can be transformed into long-term policies on the basis of coordination and perfection.
(3) Strengthen the ability of the digital economy to cope with risks: first, in response to the shortcomings exposed in COVID-19 pandemic response, large data, artificial intelligence, block chain, smart supply chain and other technologies can be used for the emergency prevention and control of major public health crises and effectively improve the efficiency and effectiveness of major pandemic prevention and control. Second, it systematically assesses the short board of the national reserve system, improves the reserve efficiency, optimizes the distribution of key material production capacity, and improves the emergency plan. Third, is to learn from the loopholes in the public health governance system, exposed by this pandemic, thoroughly investigate, and rectify similar problems in governance systems in other fields, enhance the capacity building of digital science and technology to support emergency response, and further establish and improve modern public safety emergency management system of China.
(4) Improve the digital system: first, strengthen talent support; increase cutting-edge technology research and cutting-edge talent-training; strengthen strategic scientific and technological resources, and strategic reserve capabilities in the field of COVID-19 pandemic prevention and control and public health. Second, accelerate the construction of a platform support system for the development of the digital economy. Make efforts from both the supply and demand sides to accelerate the construction and promotion of industrial internet platforms and the popularization of industrial Internet applications for SMEs; form a multilevel and systematic platform development system; promote the connection of all the industrial elements and the optimal allocation of resources. Third, accelerate the improvement of policies and provide a support system for the development of the digital economy. Establish and improve policies and regulations to adapt to the development of the digital economy, further deepen the reform of custody, promote the continuous optimization of business environments, strengthen the supervision of digital transactions, and improve the data security system.

## Data Availability Statement

The original contributions presented in the study are included in the article/supplementary material, further inquiries can be directed to the corresponding author/s.

## Author Contributions

AX, FQ, and PZ performed material preparation, data collection, and analysis. AX wrote the first draft of the manuscript. All authors contributed to the study conception and design, commented on previous versions of the manuscript, read, and approved the final manuscript.

## Funding

This study was supported by the Zhejiang Social Science Planning Project Achievements, the Research on Technology Empowerment of Digital Economy and the Digital Development Path of Manufacturing Industry (No. 22NDQN284YB).

## Conflict of Interest

The authors declare that the research was conducted in the absence of any commercial or financial relationships that could be construed as a potential conflict of interest.

## Publisher's Note

All claims expressed in this article are solely those of the authors and do not necessarily represent those of their affiliated organizations, or those of the publisher, the editors and the reviewers. Any product that may be evaluated in this article, or claim that may be made by its manufacturer, is not guaranteed or endorsed by the publisher.
